# Meta-analysis of the role of neutrophil to lymphocyte ratio in neonatal sepsis

**DOI:** 10.1186/s12879-023-08800-0

**Published:** 2023-11-28

**Authors:** Jingyang Chen, Sanaz Yasrebinia, Arshin Ghaedi, Monireh Khanzadeh, Stephan Quintin, Abeer Dagra, Rodeania Peart, Brandon Lucke-Wold, Shokoufeh Khanzadeh

**Affiliations:** 1 The First Clinical Medical College of Fujian Medical University, Fuzhou, Fujian Province China; 2 Tabriz University of Medical Sciences, Tabriz, Iran; 3 Student Research Committee, School of Medicine, Shiraz University of Medical Sciences, Shiraz, Iran; 4 Trauma Research Center, Shahid Rajaee (Emtiaz) Trauma Hospital, Shiraz University of Medical Sciences, Shiraz, Iran; 5 Geriatric & Gerontology Department, Medical School, Tehran University of Medical and Health Sciences, Tehran, Iran; 6 Department of Neurosurgery, University of Florida, Gainesville, FL 32610 USA

**Keywords:** Neutrophil to lymphocyte ratio, Neonatal sepsis, Systematic review, Meta-analysis

## Abstract

**Introduction:**

The neutrophil to lymphocyte ratio (NLR), an inflammatory biomarker, measures innate-adaptive immune system balance. In this systematic review and meta-analysis, we aim to analyze the current literature to evaluate the diagnostic role of NLR in neonatal sepsis.

**Methods:**

PubMed, Web of Science, and Scopus were used to conduct a systematic search for relevant publications published before May 14, 2022.

**Results:**

Thirty studies, including 2328 neonates with sepsis and 1800 neonates in the control group, were included in our meta-analysis. The results indicated that NLR is higher in neonates with sepsis compared to healthy controls (SMD = 1.81, 95% CI = 1.14–2.48, *P*-value < 0.001) in either prospective (SMD = 2.38, 95% CI = 1.40–3.35, *P*-value < 0.001) or retrospective studies (SMD = 0.87, 95% CI = 0.63–1.12, *P*-value < 0.001) with a pooled sensitivity of 79% (95% CI = 62–90%), and a pooled specificity of 91% (95% CI = 73–97%). Also, we found that NLR is higher in neonates with sepsis compared to those who were suspected of sepsis but eventually had negative blood cultures (SMD =1.99, 95% CI = 0.76–3.22, *P*-value = 0.002) with a pooled sensitivity of 0.79% (95% CI = 0.69–0.86%), and a pooled specificity of 73% (95% CI = 54–85%). In addition, neonates with sepsis had elevated levels of NLR compared to other ICU admitted neonates (SMD = 0.73, 95% CI = 0.63–0.84, *P* < 0.001). The pooled sensitivity was 0.65 (95% CI, 0.55–0.80), and the pooled specificity was 0.80 (95% CI, 0.68–0.88).

**Conclusion:**

Our findings support NLR as a promising biomarker that can be readily integrated into clinical settings to aid in diagnosing neonatal sepsis. As evidenced by our results, restoring balance to the innate and adaptive immune system may serve as attractive therapeutic targets. Theoretically, a reduction in NLR values could be used to measure therapeutic efficacy, reflecting the restoration of balance within these systems.

## Introduction

Neonatal sepsis is a bloodstream infection that affects newborn infants under 28 days. It is a leading cause of morbidity and mortality in these children [1, 2]. There are around 2,200 instances of neonatal sepsis per 100,000 live births, with a death rate of 11 to 19 percent [3]. The mortality rate varies depending on factors such as birth weight, with rates of 50% in newborns with a birth weight of less than 1,500 g, 23.8% in those with a birth weight of 1,500–2,500 g, and 18.2% overall [4]. Neonatal sepsis has also several long-term health outcomes such as neurodevelopmental disabilities (like cerebral palsy, visual or hearing impairments, and cognitive problems), respiratory complications (like chronic lung disease and bronchopulmonary dysplasia), nutritional and growth issues, and immunological dysfunction [5, 6].

Early-onset sepsis (EOS) and late-onset sepsis (LOS) are two types of neonatal sepsis. Sepsis in neonates that occurs before 72 h of life (some experts use 7 days) is referred to as EOS, while sepsis that occurs after 72 h of life is referred to as LOS [7, 8].

The spread of infections from the female genitourinary system to the infant or fetus is the most common cause of EOS [1, 2, 7, 9]. These infections can contaminate the amniotic fluid or may ascend the vaginal canal, cervix, and uterus. As they pass through the vaginal canal in pregnancy or upon delivery, neonates can become contaminated. Group B Streptococci (GBS) and E. coli are common bacterial infections associated with EOS [1, 2].

In addition, birth before 37 weeks and protracted rupture of membranes are all maternal variables that enhance the risk of neonatal sepsis [3]. Delayed treatment of newborn sepsis is linked to a variety of adverse outcomes, including persistent lung illness and neurodevelopmental concerns [1, 2]. Overuse of antibiotics, for prophylactic treatment in sepsis prevention, on the other hand, can raise the risk of severe candidiasis and multidrug-resistant organisms [1, 2, 7, 9].

In contrast, LOS is commonly caused by pathogens in the environment following delivery, often originating from caregivers or healthcare workers [1, 2, 7, 8]. Occasionally, it may stem from a delayed manifestation of an infection passed from the mother. Infants who undergo invasive procedures or have intravascular catheters are more susceptible to LOS [7]. Premature babies are at a greater risk of infection or sepsis and mortality than full-term infants [2, 9]. EOS and LOS have some differences in terms of symptoms. EOS represent as respiratory distress, hypoglycemia, apnea, hypoglycemia, and lethargy [10, 11]. On the other hand LOS represent as fever, poor feeding, lethargy, and respiratory distress [12, 13].

Research indicates that E. coli is related to higher mortality rates compared to GBS [8]. A lower mortality rate has also been observed with early treatment of clinically suspected neonates [10].

The immature immune system is the primary cause of increased neonatal sepsis susceptibility [10, 14, 15]. Neutrophils, macrophages, and T lymphocytes cannot carry out a complete inflammatory response in newborns due to their immature function. Furthermore, newborns have a restricted quantity of immunoglobulins at birth [10, 14–16]. The premature infant’s limited time in the uterus reduces the transfer of immune globulins to the fetus [1, 2].

Several clinical investigations have recently established the efficacy of the neutrophil to lymphocyte ratio (NLR) in predicting newborn sepsis [17–46]. This paper provides a systematic review and meta-analysis of these studies.

## Methods

### Search strategy

The preferred reporting items for systematic reviews and meta-analyses (PRISMA) standard was followed for this meta-analysis. From conception through May 14, 2022, a systematic review was undertaken using ISI Web of Science, PubMed, and Scopus. The following key phrases were used in search strategies: ((neutrophil AND lymphocyte AND ratio) OR NLR) AND (Neonat* or infant) AND (sepsis OR septic OR bacteremia). There were no date or language restrictions. Furthermore, potential meta-analyses and reviews were manually reviewed to find any further relevant articles that would be appropriate for this study.

### Inclusion and exclusion criteria

According to the PICOS (Participants, Intervention, Comparison, Outcomes, and Study Design) framework, two researchers independently discovered and chose the studies:

- Population: Newborns with sepsis
- Intervention: NLR
- Control: newborns suspected of having sepsis who subsequently had negative blood cultures OR healthy newborns OR newborns admitted to the intensive care unit (ICU)
- Outcomes: NLR’s ability as a diagnostic tool for neonatal sepsis
- Study Design. case-control or cross-sectional publications

Only the most recent or thorough studies were chosen to represent the reduplicative patient group.

### Data extraction

Two researchers separately gathered data from the included papers. We gathered the following information:

1) general characteristics: country, publication year, first author, study subjects, study design (retrospective or prospective); 2) sample size in case and control groups; 3) NLR values; 4) the number of controls and cases and their NLR values; 5) type of control group (neonates admitted to the ICU, healthy neonates, sepsis suspected neonates but with final negative blood culture results; 6) sepsis type: LOS, EOS, both; 7) cut-off value, specificity, and sensitivity of NLR. We contacted the corresponding authors of included articles when further information was needed.

### Quality assessment

The quality evaluation of included papers was done with the assistance of the Newcastle-Ottawa Scale (NOS). This scale consists of three parts: comparability, selection, and outcome.

### Statistical analysis

With the help of STATA application version 12.0 (StataCorp LP, College Station, TX, USA), the meta-analysis of the correlation between neonatal sepsis and NLR was conducted. NLR differences between cases and controls were measured using Standardized mean difference (SMD) with a 95% confidence interval (CI). We reported SMD to accommodate the differences in NLR measurement techniques across various studies. 95% CI was reported to show the likely range of effect sizes supported by the findings. I^2^ metric and Q chi-square test were used to quantify the heterogeneity among studies. When the *P* value was lower than 0.05, and I^2^ was more than 50%, we assumed considerable heterogeneity exists. If we detected significant heterogeneity, we used a random-effect model; otherwise, we used a fixed-effect model. To determine the diagnostic value of NLR for sepsis in newborns, we created a summary receiver operating characteristic (SROC) curve by the “metandi” command. The initial construction of the SROC curve involves plotting the sensitivity (which represents true positivity) and the complement of specificity (1 - specificity) for each study. This curve is widely employed to gauge the accuracy of diagnostic tests. Essentially, the closer the ROC curve gets to the upper left corner of the graph, the higher the test’s accuracy, as this position corresponds to a sensitivity of 1 and a false positive rate of 0 (equivalent to a specificity of 1). Ultimately, we used Egger’s test and funnel plot (visual inspection) to detect possible publication bias. In Egger’s test, we use linear regression to assess the association between the standardized effect estimates and the standard error (SE); so, *P*-Value < 0.05 can be interpreted as a significant publication bias across studies.

## Results

### Search and selection of literature

**Fig. 1:**
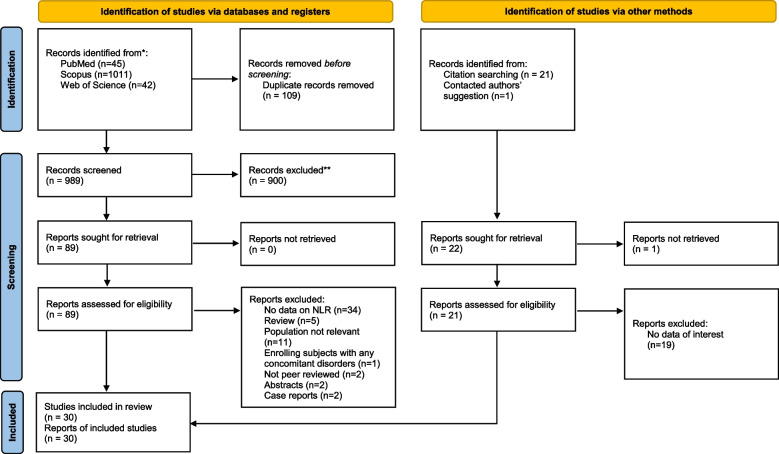
PRISMA 2020 Flow diagram for new systematic reviews which includes searches of databases, registers and other sources

The database search and the manual search of the article citation list turned up 1120 records. Thirty studies were included in the systematic review and meta-analysis [17–46] after duplicates and irrelevant records were removed. Included studies had a total of 2328 neonates with sepsis and 1800 neonates in the control group, of which 902 were healthy neonates, 271 were neonates who were initially suspected of having sepsis but ultimately had negative blood cultures, and 627 were other neonates admitted to the ICU. The PRISMA flow diagram, shown in Fig. 1, describes the inclusion and exclusion processes in detail.

### Characteristics of included studies

Table 1 displays the methodological and characteristic qualities of these publications. The overall study quality ranged from 6 to 8 stars. Thirty studies were included in our systematic review and meta-analysis [17–46]. Of them, 15 studies had a control group of healthy neonates [19, 24–27, 29, 30, 32, 33, 35–39, 46], five studies had a control group of neonates who were suspected of sepsis, but eventually had negative blood cultures [20, 28, 40, 41, 44], and ten studies included other ICU admitted neonates as the control group [17, 18, 21–23, 31, 34, 42, 43, 45]. Twenty studies were prospective [17–25, 27, 29, 30, 32, 35–38, 41, 42, 44] and 10 studies were retrospective [26, 28, 31, 33, 34, 39, 40, 43, 45, 46]. Nine studies included neonates with early-onset sepsis [17, 24, 25, 27, 31, 35, 37, 44, 46] and five included neonates with late-onset sepsis [20, 26, 28, 29, 43]. Other studies considered both types of sepsis.

**Table 1:** General characteristics of included studies

First author	Year	Country	Design	Control group	Type of sepsis	cut-off point of NLR	Sensitivity	Specificity	Cases		Controls		NOS score
									N	NLR	N	NLR	
Can et al. [24]	2017	Turkey	P	Healthy neonates	EOS	6.76	97	100	78	2.88 ± 0.16	44	0.21 ± 0.12	6
Khattab et al. [32]	2018	Egypt	P	Healthy neonates	Both types	1.06	82	83	60	1.97 ± 1.09	30	0.95 ± 0.48	6
Omran et al. [38]	2018	Egypt	P	Healthy neonates	Both types	2.7	80	57	35	2.90 ± 1.70	35	1.60 ± 0.40	7
Ghrahani et al. [27]	2019	Indonesia	P	Healthy neonates	EOS	_	_	_	22	1.85 ± 2.07	31	2.48 ± 2.06	6
Mahmoud et al. [35]	2019	Egypt	P	Healthy neonates	EOS	0.1	67	99	40	0.80 ± 1.10	40	0.08 ± 0.30	7
Yalinbash et al. [26]	2019	Turkey	R	Healthy neonates	LOS	1.18	69	67	48	2.65 ± 2.72	60	1.22 ± 1.25	7
Israr et al. [30]	2020	Pakistan	P	Healthy neonates	Both types	1.39	65	63	19	1.91 ± 0.93	41	1.54 ± 0.99	6
MM et al. [37]	2020	Cairo	P	Healthy neonates	EOS	2.52	97	100	30	3.13 ± 0.49	30	0.38 ± 0.18	6
Chen et al. [25]	2021	China	P	Healthy neonates	EOS	2.01	83	76	63	2.94 ± 2.00	188	1.68 ± 1.14	7
Hibbert et al. [29]	2021	Australia	P	Healthy neonates	LOS	_	_	_	43	1.40 ± 0.80	76	1.80 ± 0.20	7
Zhang et al. [46]	2021	China	R	Healthy neonates	EOS	3.16	77	78	74	4.34 ± 1.77	50	2.49 ± 0.90	7
Mao et al. [36]	2021	China	P	Healthy neonates	Both types	_	_	_	90	5.38 ± 2.21	88	1.22 ± 0.30	7
Nady et al. [19]	2021	Egypt	P	Healthy neonates	Both types	1.66	97	90	60	3.06 ± 2.41	60	1.39 ± 1.32	7
Panda et al. [39]	2021	India	R	Healthy neonates	Both types	1.7	68	46	41	3.88 ± 1.78	52	2.34 ± 1.90	6
Kurt et al. [33]	2021	Turkey	R	Healthy neonates	Both types	4.79	9	99	57	1.95 ± 2.12	77	0.81 ± 0.87	6
Goldberg et al. [28]	2021	USA	R	Suspected sepsis	LOS	1.00	90	70	33	4.30 ± 0.07	72	0.90 ± 0.60	8
Ozdemir et al. [20]	2017	Turkey	P	Suspected sepsis	LOS	1.77	73	78	52	3.69 ± 3.00	75	1.56 ± 1.83	7
Ruslie et al. [40]	2018	Indonesia	R	Suspected sepsis	Both types	9.4	62	67	52	14.24 ± 7.14	42	6.29 ± 1.41	6
Sumitro et al. [41]	2021	Indonesia	P	Suspected sepsis	Both types	2.12	81	42	52	4.20 ± 3.20	52	3.40 ± 2.50	7
Wilar et al. [44]	2019	Indonesia	P	Suspected sepsis	EOS	1.24	83	93	90	2.82 ± 2.29	30	0.82 ± 0.32	6
Abdelmoktader et al. [17]	2020	Egypt	P	Other ICU-admitted neonates	EOS	1.75	70	76	71	3.10 ± 3.50	29	1.70 ± 0.90	6
Akhmaltdinova et al. [18]	2021	Kazakhstan	P	Other ICU-admitted neonates	Both types	1.00	47	95	26	1.09 ± 0.61	20	0.54 ± 0.42	6
Ashour et al. [21]	2022	Egypt	P	Other ICU-admitted neonates	Both types	_	_	_	100	2.69 ± 3.30	50	1.20 ± 0.80	7
Awad et al. [22]	2022	Egypt	P	Other ICU-admitted neonates	Both types	1.46	82	55	18	3.20 ± 2.30	18	1.30 ± 0.60	6
Buyukeren et al. [23]	2021	Turkey	P	Other ICU-admitted neonates	Both types	_	_	_	77	3.55 ± 3.79	131	1.53 ± 0.71	6
Karabulut et al. [31]	2020	Turkey	R	Other ICU-admitted neonates	EOS	1.42	88	84	30	3.16 ± 1.72	30	0.99 ± 0.75	6
Li et al. [34]	2020	China	R	Other ICU-admitted neonates	Both types	1.62	51	75	737	1.85 ± 1.64	188	1.00 ± 0.61	8
Tang et al. [42]	2017	China	P	Other ICU-admitted neonates	Both types	_	_	_	16	2.90 ± 2.10	26	1.96 ± 0.40	6
Varal et al. [43]	2020	Turkey	R	Other ICU-admitted neonates	LOS	1.57	68	82	76	3.20 ± 2.50	40	1.40 ± 0.20	6
Yorulmaz et al. [45]	2018	Turkey	R	Other ICU-admitted neonates	Both types	_	_	_	138	2.07 ± 1.28	95	1.03 ± 0.77	7

*N* Number, *NLR* Neutrophil to lymphocyte ratio, *LOS* Late-onset sepsis, *EOS* Early-onset sepsis, *NOS* The Newcastle-Ottawa Quality Assessment Scale, *R* Retrospective, *P* Prospective, *ICU* Intensive care unit

### The differences in NLR levels between neonates with sepsis and healthy controls

**Fig. 2:**
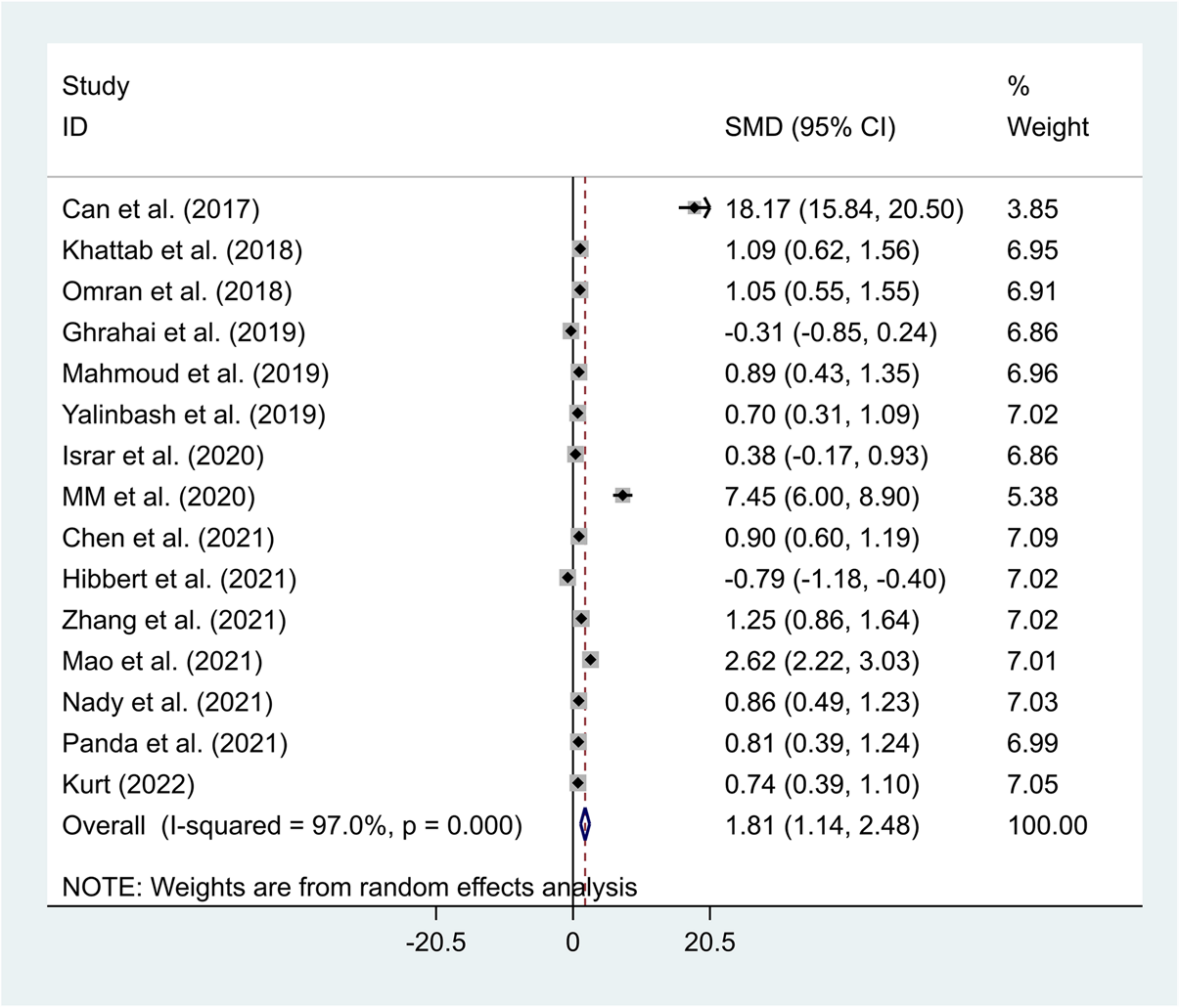
Meta-analysis of differences in NLR level between neonates with sepsis and healthy controls

Considering the statistical heterogeneity across studies, the pooled meta-analysis was conducted using a random-effects model (I^2^ = 97.0%, *P*-value < 0.001). The results indicated that neonates with sepsis had elevated levels of NLR compared to healthy controls (SMD = 1.81, 95% CI = 1.14–2.48, *P*-value < 0.001) (Fig. 2).

**Fig. 3:**
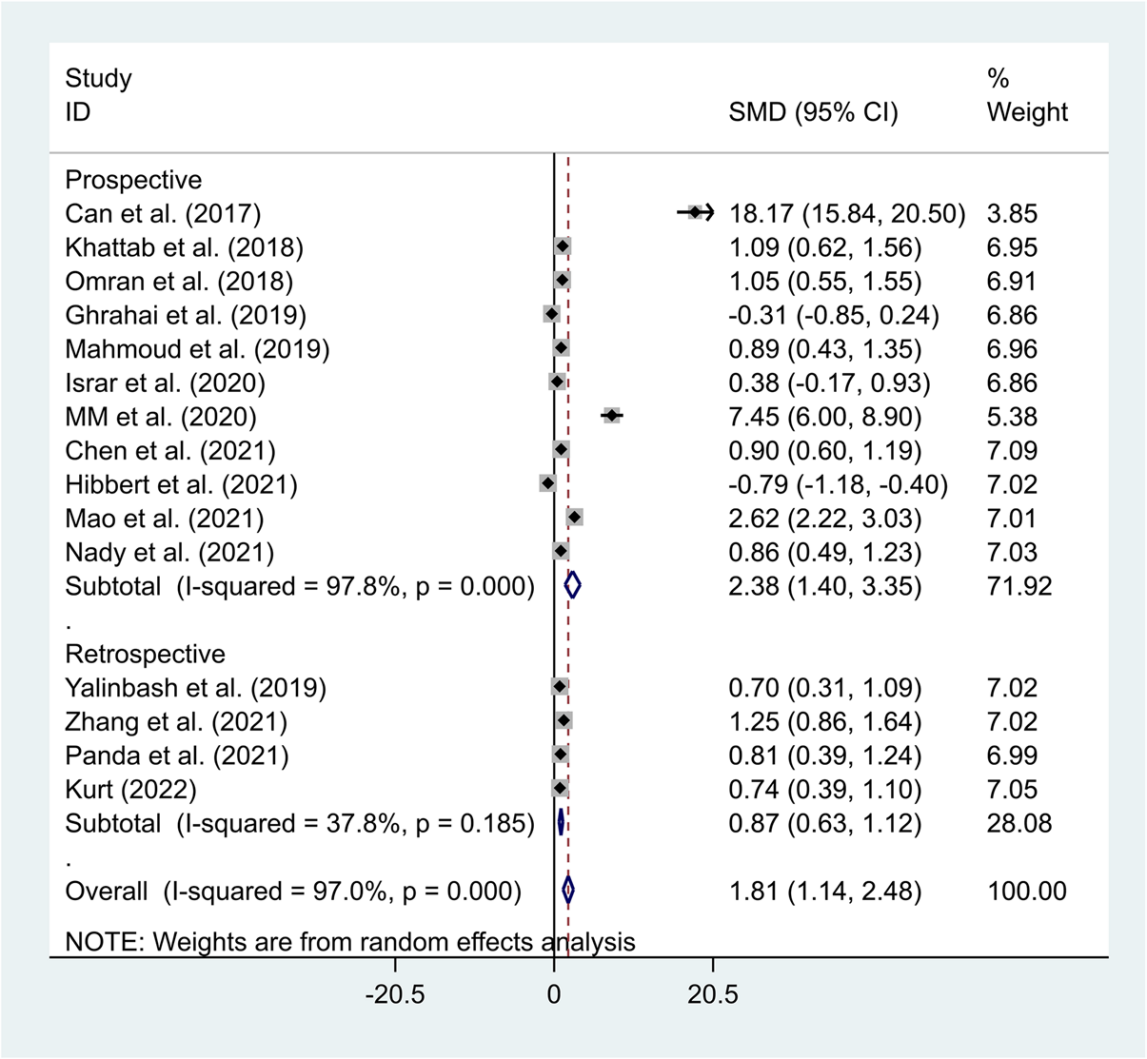
Subgroup analysis of differences in NLR level between neonates with sepsis and healthy controls according to study design

Further subgroup analysis stratified by study design indicated that neonates with sepsis had elevated levels of NLR compared to healthy controls in either prospective (SMD = 2.38, 95% CI = 1.40–3.35, *P*-value < 0.001) or retrospective studies (SMD = 0.87, 95% CI = 0.63–1.12, *P*-value < 0.001) (Fig. 3).

**Fig. 4:**
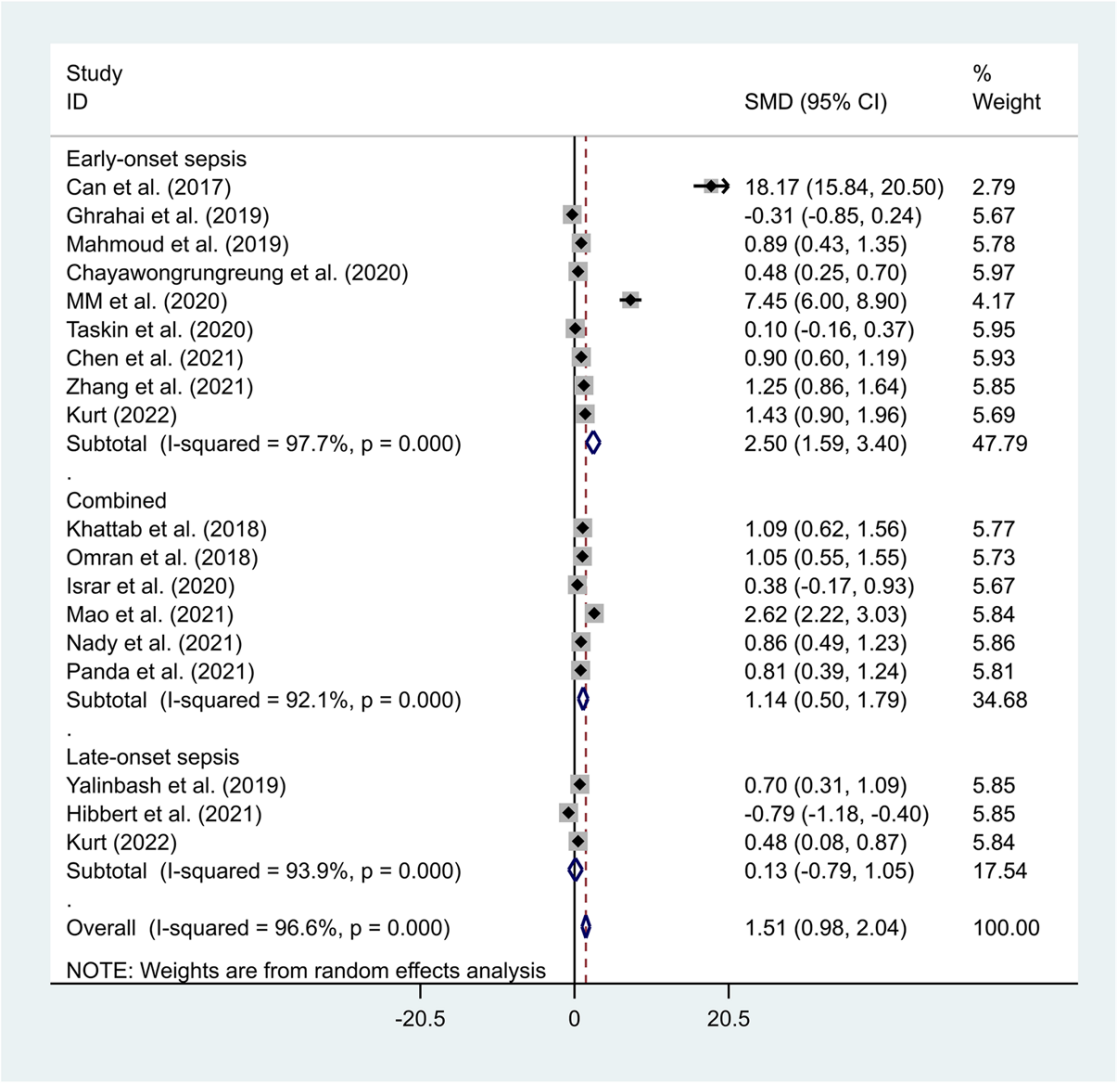
Subgroup analysis of differences in NLR level between neonates with sepsis and healthy controls according to the type of sepsis

Another subgroup analysis of the type of sepsis indicated that neonates with early-onset sepsis had elevated levels of NLR compared to healthy controls (SMD = 2.50, 95% CI = 1.59–3.40, *P*-value < 0.001). However, there was no difference in NLR level between neonates with late-onset sepsis and healthy controls (SMD = 0.13, 95% CI = -0.79–1.05, *P*-value = 0.78) (Fig. 4).

### Diagnostic value of NLR for differentiating between neonates with sepsis and healthy controls

**Fig. 5:**
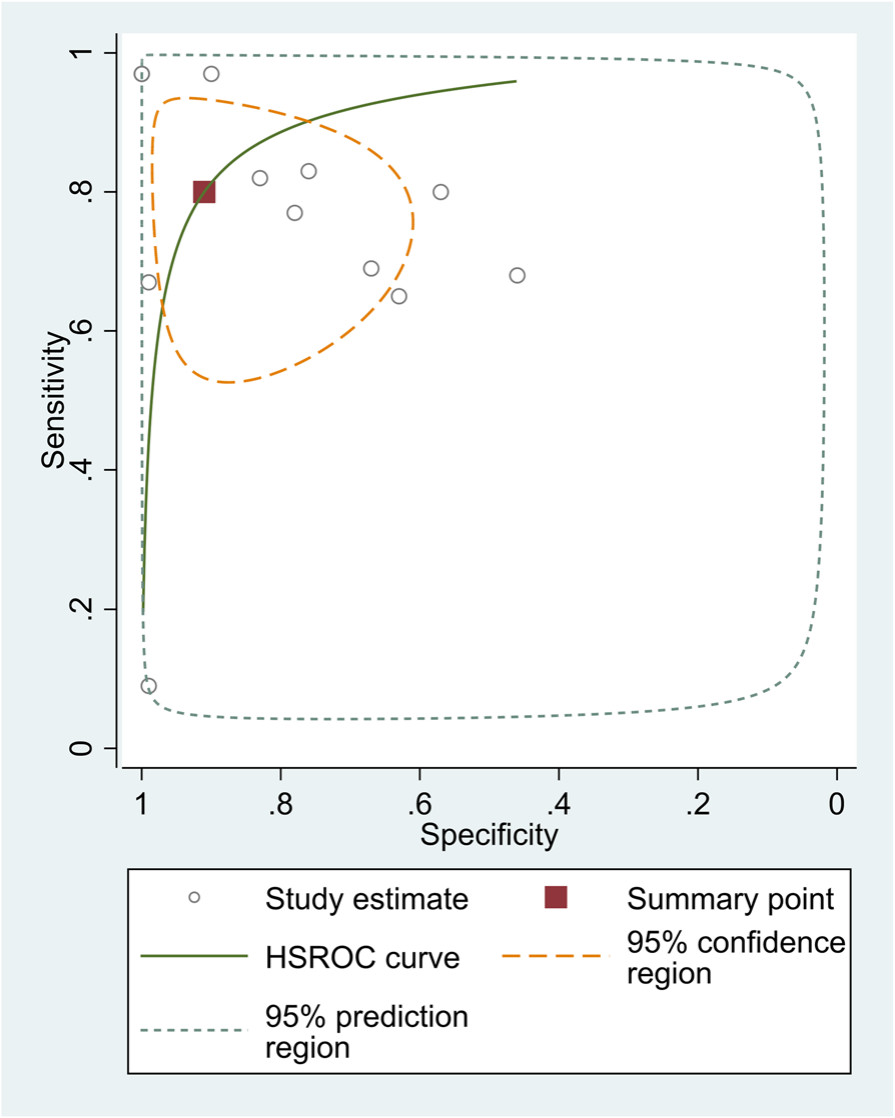
SROC curve of included studies in the meta-analysis of differences in NLR level between neonates with sepsis and healthy controls

The pooled sensitivity of 12 studies was 0.79 (95% CI, 0.62–0.90), and the pooled specificity was 0.91 (95% CI, 0.73–0.97). The pooled positive likelihood ratio, negative likelihood ratio, DOR of NLR were 8.88 (95%CI = 2.65–29.80), 0.21 (95%CI = 0.10–0.45), and 40.42 (95%CI = 7.48–218.38), respectively (Fig. 5).

### The differences in NLR levels between neonates with sepsis and those who were suspected of sepsis but eventually had negative blood cultures

**Fig. 6:**
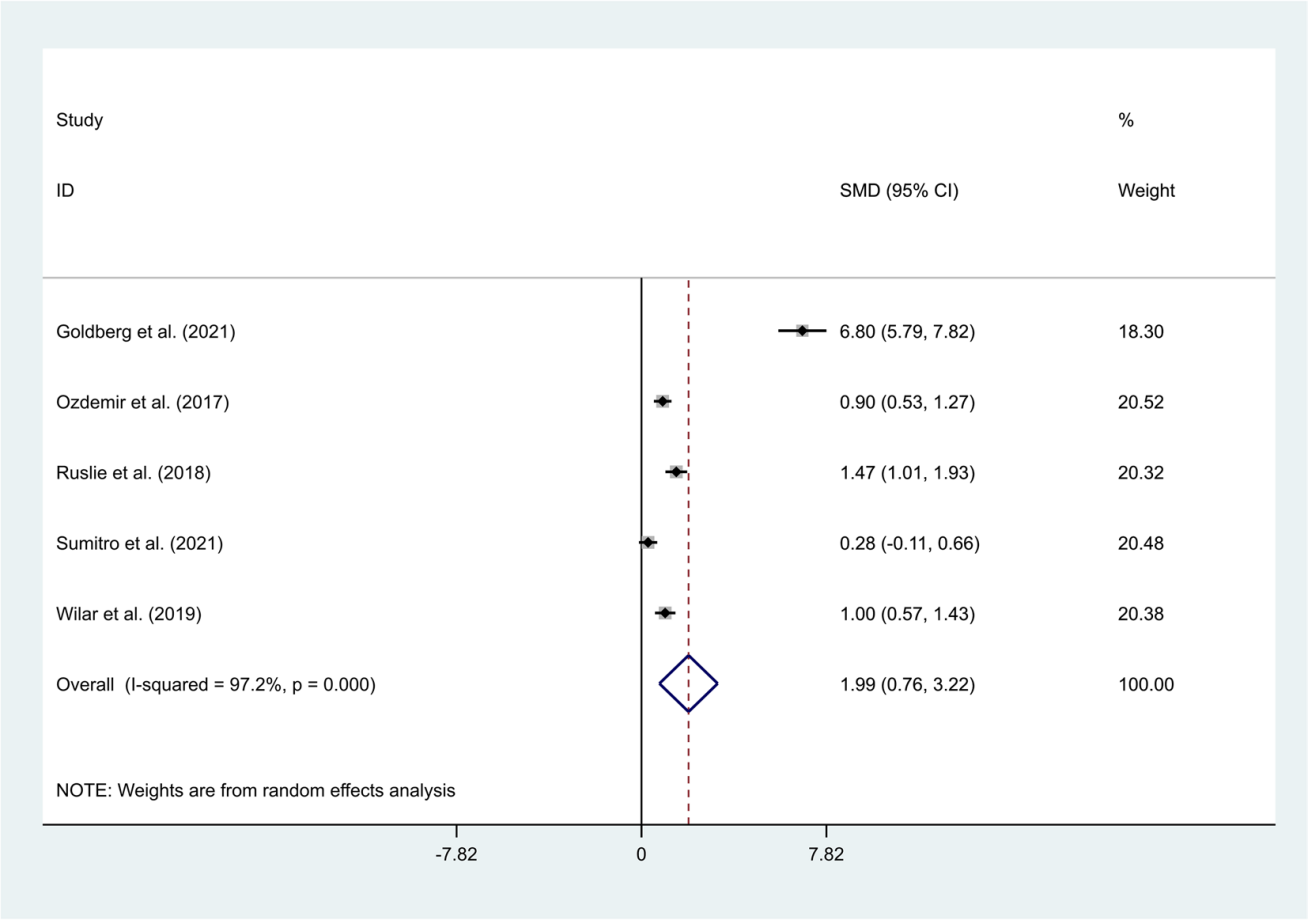
Meta-analysis of differences in NLR level between neonates with confirmed sepsis and those with suspected sepsis

The random-effects model was applied to the pooled meta-analysis, as statistical heterogeneity existed among studies (I^2^ = 97.2%, *P*-value < 0.001). We found that neonates with sepsis had elevated levels of NLR compared to those who were suspected of sepsis, but eventually had negative blood cultures (SMD = 1.99, 95% CI = 0.76–3.22, *P*-value = 0.002) (Fig. 6).

**Fig. 7:**
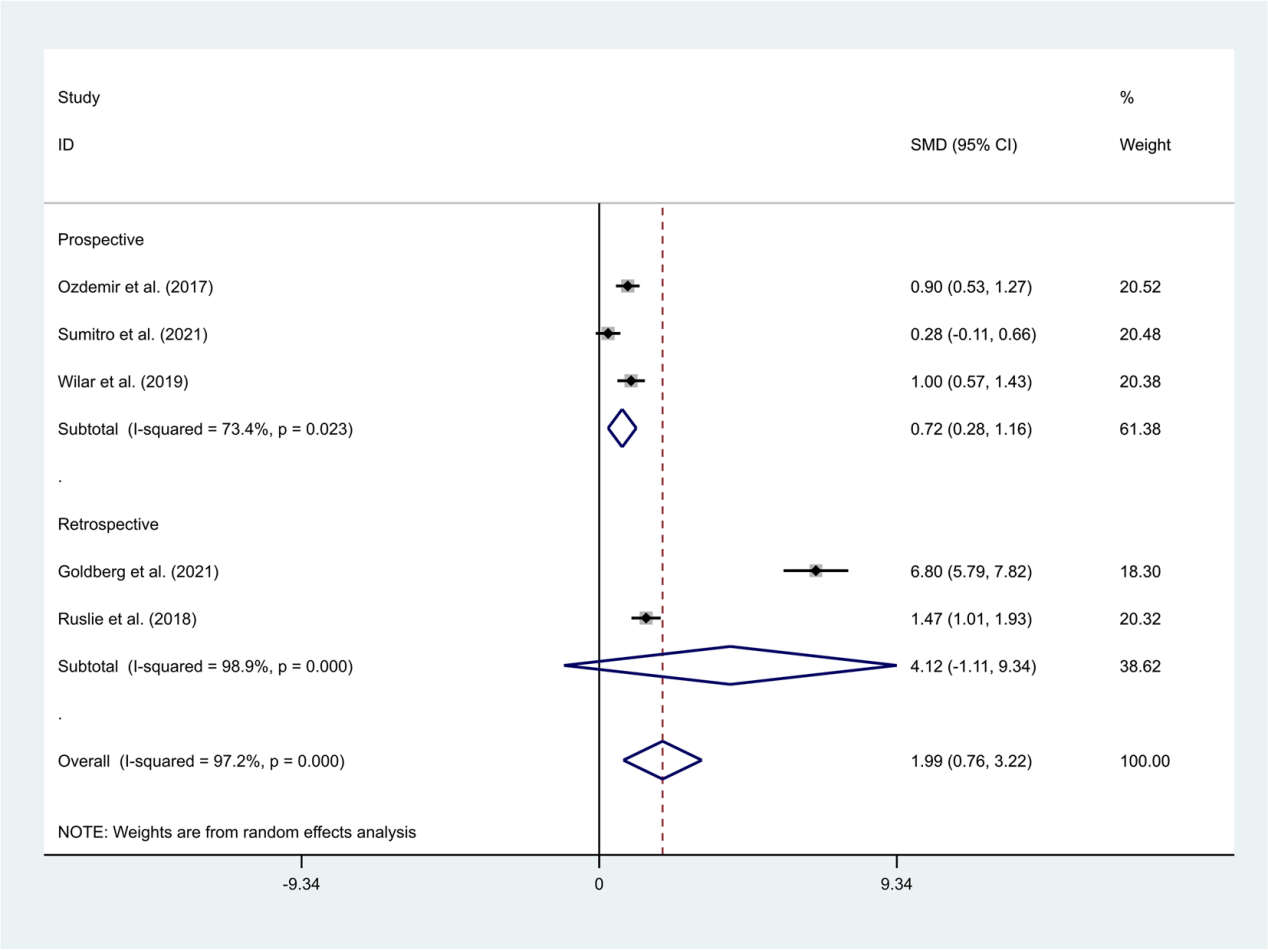
Subgroup analysis of differences in NLR level between neonates with confirmed sepsis and those with suspected sepsis, according to study design

In the subgroup analysis according to study design, we found that neonates with sepsis had elevated levels of NLR compared to those who were suspected of sepsis, but eventually had negative blood cultures in prospective studies (SMD = 0.72, 95% CI = 0.28–1.16, *P*-value = 0.001), but not in retrospective studies (SMD = 4.12, 95% CI = -1.11–9.34, *P*-value = 0.122) (Fig. 7).

### Diagnostic value of NLR for differentiating between neonates with sepsis and those who were suspected of sepsis, but eventually had negative blood cultures

**Fig. 8:**
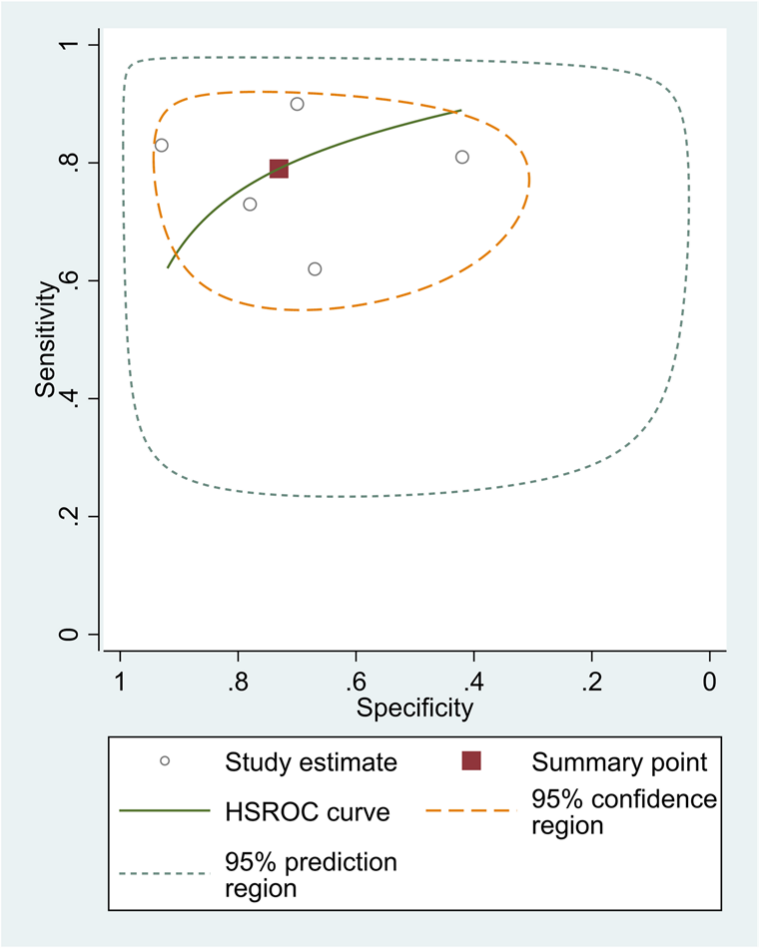
SROC curve of included studies in the meta-analysis of differences in NLR level between neonates with confirmed sepsis and those with suspected sepsis

The pooled sensitivity of five studies was 0.79 (95% CI, 0.69–0.86), and the pooled specificity was 0.73 (95% CI, 0.54–0.85). The pooled positive likelihood ratio, negative likelihood ratio, DOR of NLR were 2.93 (95%CI = 1.58–5.41), 0.28 (95%CI = 0.17–0.46), and 10.20 (95%CI = 3.75–27.70), respectively (Fig. 8).

### The differences in NLR levels between neonates with sepsis and other ICU admitted neonates

**Fig. 9:**
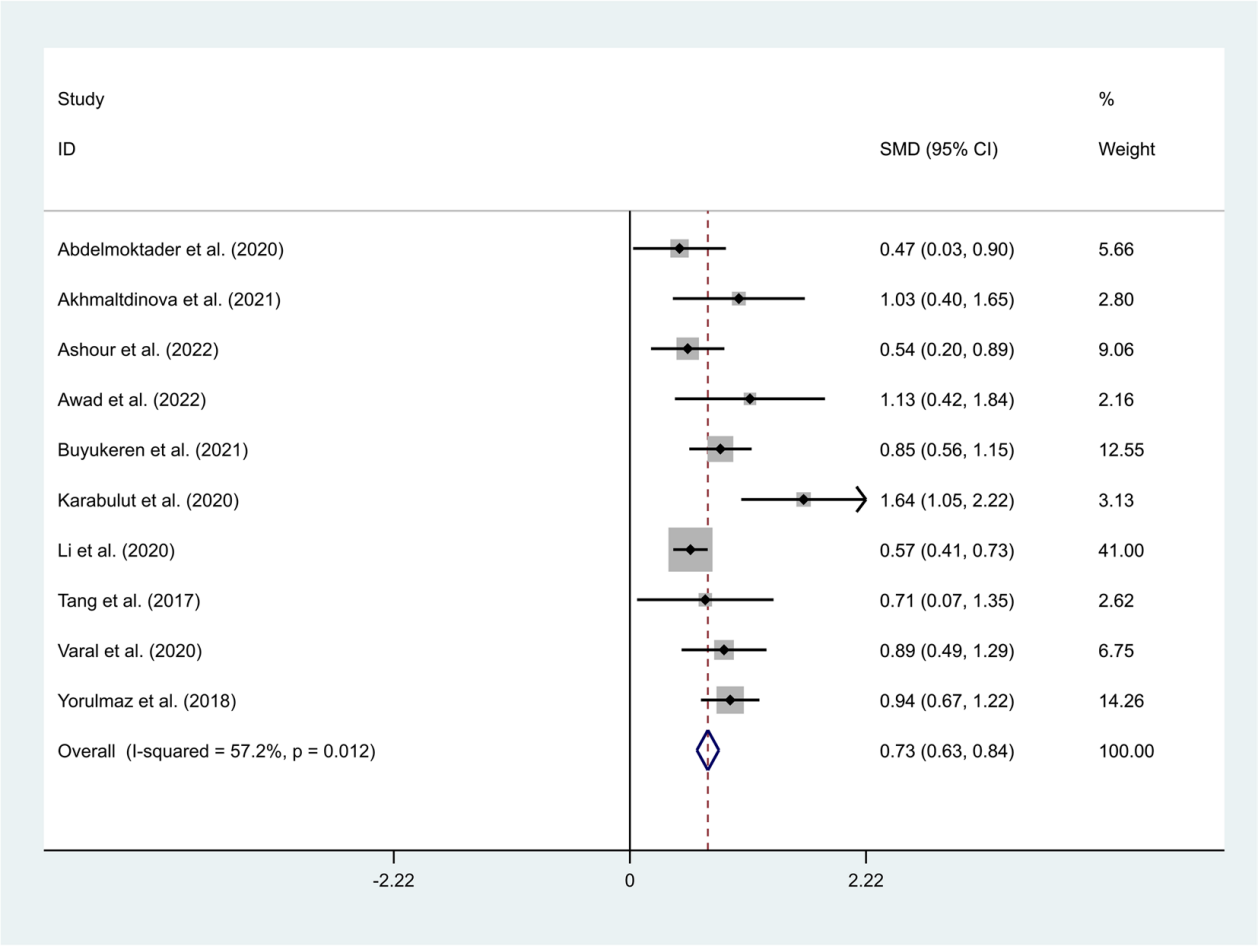
Meta-analysis of differences in NLR level between neonates with sepsis and other ICU admitted neonates

A fixed-effects model was applied to t pool the data of included studies (I^2^ = 57.2%, *P*-value < 0.01). Neonates with sepsis had elevated levels of NLR compared to other ICU admitted neonates (SMD = 0.73, 95% CI = 0.63–0.84, *P* < 0.001) (Fig. 9).

**Fig. 10:**
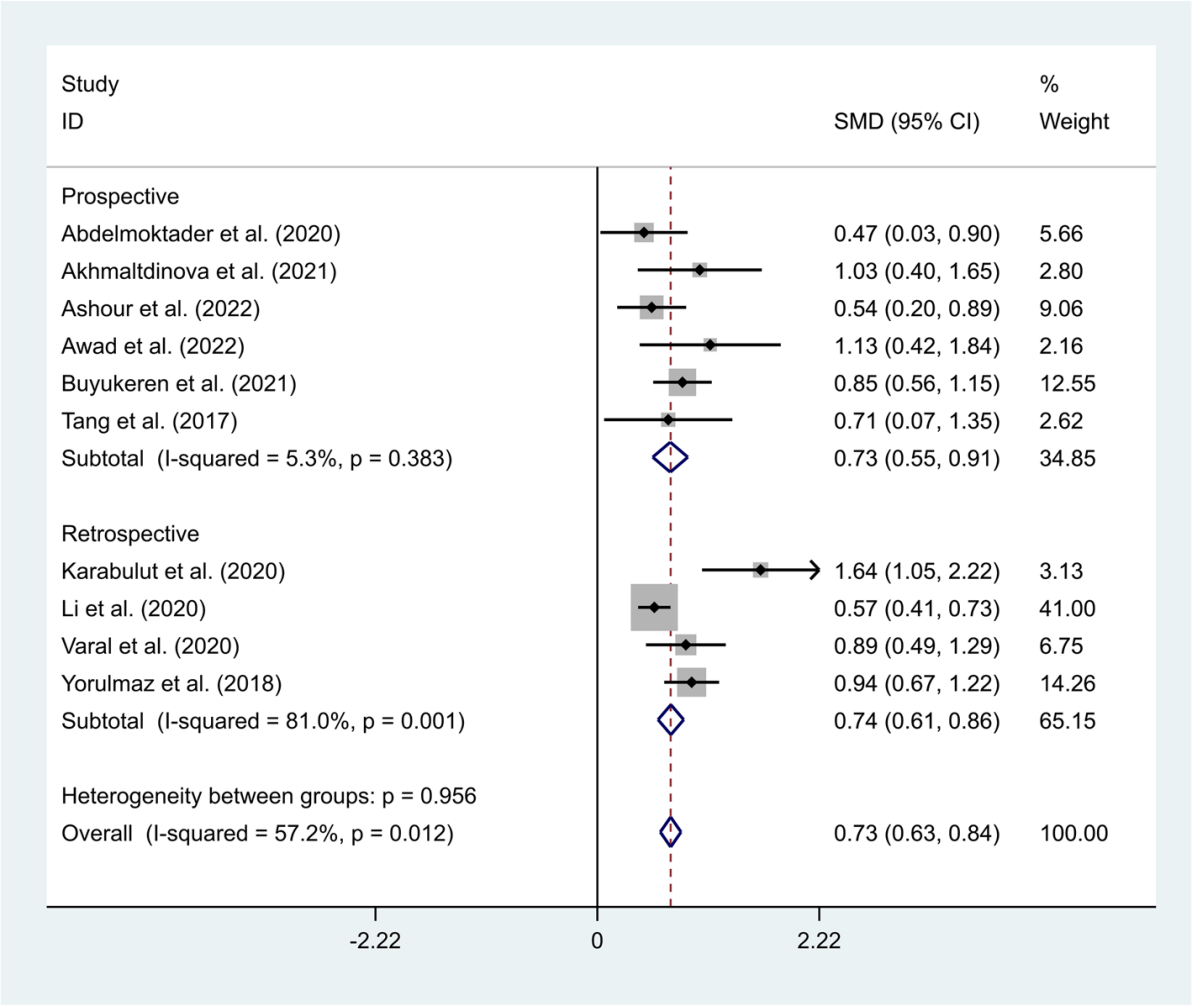
Subgroup analysis of differences in NLR level between neonates with sepsis and other ICU admitted neonates, according to study design

Then, we conducted a subgroup analysis according to the study design. The results showed that neonates with sepsis had elevated levels of NLR compared to Other ICU admitted neonates in either prospective (SMD = 0.73, 95% CI = 0.55–0.91, *p* < 0.001) or retrospective studies (SMD = 0.74, 95% CI = 0.61–0.86, *p* < 0.001) (Fig. 10).

### Diagnostic value of NLR for differentiating between neonates with sepsis and other ICU admitted neonates

**Fig. 11:**
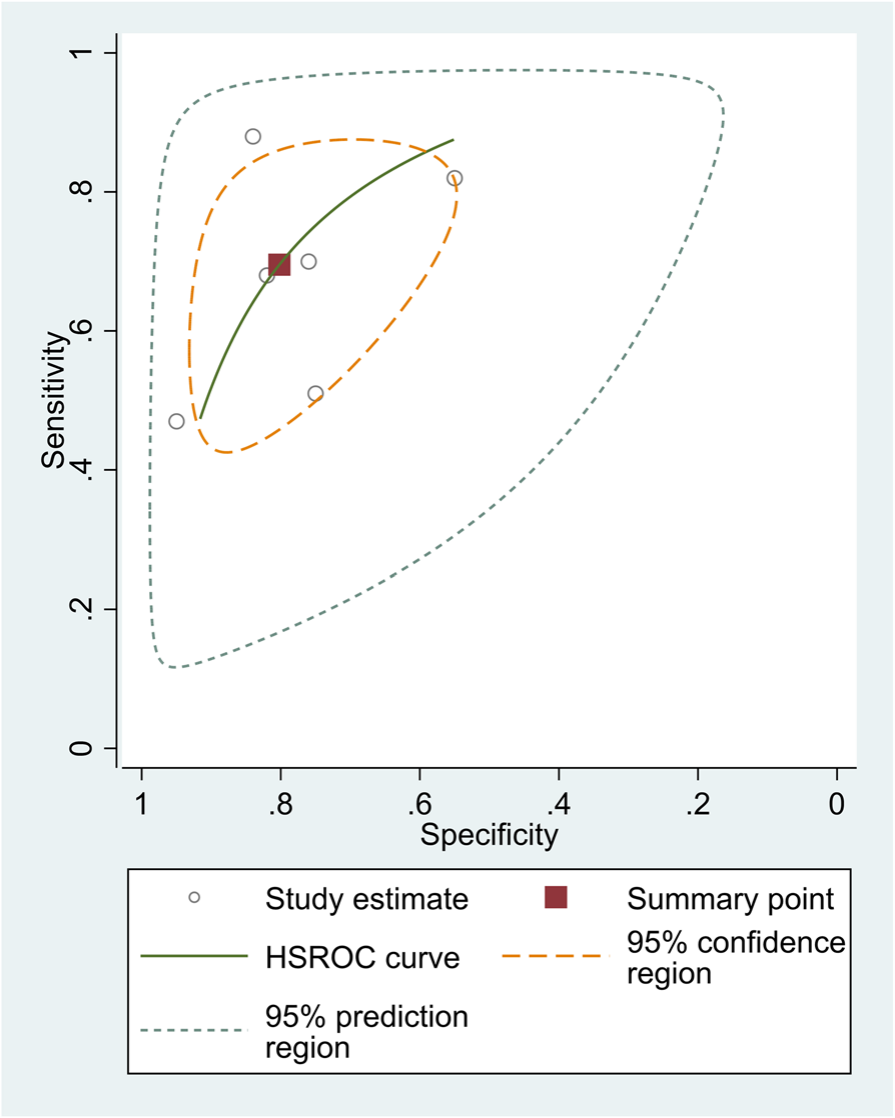
SROC curve of included studies in the meta-analysis of differences in NLR level between neonates with sepsis and other ICU admitted neonates

The pooled sensitivity of five studies was 0.65 (95% CI, 0.55–0.80), and the pooled specificity was 0.80 (95% CI, 0.68–0.88). The pooled positive likelihood ratio, negative likelihood ratio, DOR of NLR were 3.51(95%CI = 2.22–5.53), 0.38 (95%CI = 0.26–0.54), and 9.23 (95%CI = 4.90–17.39), respectively (Fig. 11).

### Publication bias

**Fig. 12:**
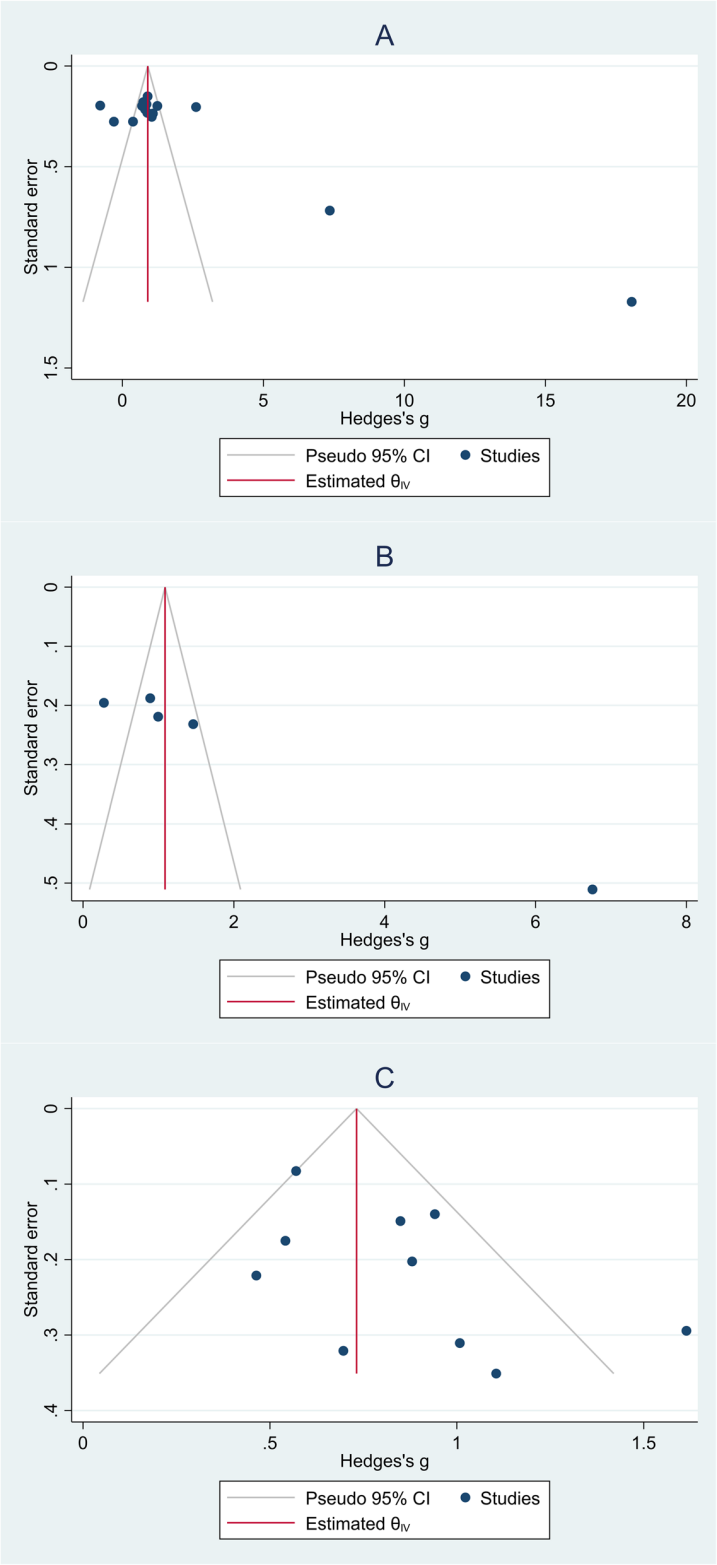
Funnel plots assessing publication bias among studies; **A** studies with a control group of healthy controls; **B** studies with a control group of neonates who were suspected of sepsis; **C** studies with a control group of other ICU-admitted neonates

As seen in Fig. 12, there was some indication of publication bias among studies with a control group of healthy neonates (Egger’s test *P*-value < 0.001) and neonates who were suspected of sepsis (Egger’s test *P*-value < 0.001). However, studies with a control group including other ICU-admitted neonates had no publication bias (Egger’s test *P*-value = 0.08).

## Discussion

Neonatal sepsis can manifest itself clinically in a variety of ways, including feeding intolerance, temperature instability, tachycardia, pneumonia, and respiratory distress [10, 14–16]. Because these symptoms are similar to non-infectious diseases, newborn sepsis is difficult to identify clinically. Some neonates with bacteremia might even have no symptoms and present with a regular physical examination [2, 9]. This emphasizes the importance of decisive tests with quantitative measures for diagnosing sepsis. Blood culture as a diagnostic marker is currently the gold standard for diagnosing sepsis in neonates, although it has drawbacks such as a long waiting period and the risk of contamination [2]. CBC with differential and C-reactive protein (CRP) are additional crucial lab tests to get and are routinely collected on a serial basis; however, these indices are weak at diagnosing newborn sepsis and are better suited for ruling it out [2]. These flaws have emphasized the necessity for a neonatal sepsis marker that can be tested rapidly and easily. As a measure of newborn sepsis, neutropenia has a higher specificity than neutrophilia [2, 47]. An elevated immature to total neutrophil (I/T) ratio of more than 0.27 has a very high negative predictive accuracy (99%) but a poor positive predictive value (25%) because it can be elevated in up to 50% of uninfected infants [2]. These counts can be erroneously raised, especially after a baby is born. However, several clinical investigations have recently established the efficacy of NLR in predicting neonatal sepsis [17–46]. Pooling the data of these studies indicated elevated levels of NLR in septic neonates versus healthy controls, found in both retrospective and prospective studies, lend to the accuracy of NLR for diagnosis of sepsis. Sub-group analysis showed this association was primarily seen in the context of EOS and combined studies rather than LOS, whereas studies of NLR in LOS were limited in number. The use of healthy controls as a comparator for indicators of neonatal sepsis may be less clinically relevant to studies comparing acutely ill septic neonates to those which are acutely ill but non-septic. Although not shown by limited retrospective studies, the findings in prospective studies of elevated NLR in septic versus neonates with suspected sepsis may indicate the negative predictive value of elevated NLR in neonatal sepsis. Additionally, findings from both prospective and retrospective cohort studies showed that elevated NLR distinguished neonatal sepsis among other ICU-admitted neonates. These findings are important because hospital admission and subsequent environmental stressors may represent confounders if not controlled for in the study design. Previous studies outlined in this paper showed a potential for false positivity or negativity when using clinical presentation or blood-based biomarkers to differentiate sepsis from non-infectious acute illnesses in neonates [2, 9]. In addition, NLR’s accuracy in diagnosing sepsis is an important finding.

### Limitations

Significant to consider in the interpretation of currently available studies, including healthy controls or controls with suspected sepsis, is the potential for small study/publication bias indicated here via Eggers linear regression analysis. A high degree of statistical heterogeneity is shown in combined, and subgroup analysis, except for studies of septic versus other ICU admitted neonates. Heterogeneity in these studies may be partially attributed to the differing diagnostic threshold for sepsis as well as differences in sample handling. Similar heterogeneity is seen across other sepsis biomarker studies, including CRP and procalcitonin [48, 49]. Sub-group analysis helps to reduce some of the heterogeneity seen in these studies and identify potential study design flaws such as inadequate comparators. The use of comparators with suspected sepsis or that are admitted to the ICU may represent a more clinically relevant approach. However, current studies lack precision, and acutely ill comparators may bring additional confounders, contributing to study heterogeneity. Attenuating this effect may require large multicenter prospective studies.

A challenge to using hematological and acute phase markers for accurate diagnosis of sepsis is the variability of immune response in the context of comorbid conditions, gestational age, post-natal age, timing of sepsis onset, and the nature of infectious agents. Coupling this pathophysiologic variability with diagnostic and procedural differences in clinical practice make real-world use of any single biomarker challenging. While markers such as CRP and I/T ratio have been shown independently to have high sensitivities, these findings depend on timing from the onset of sepsis, either lacking accuracy early on or losing accuracy later in the course of sepsis [8, 50]. The findings in this review support the use of NLR to diagnose neonatal sepsis among the limitations of current biomarkers. The use of CRP and NLR as combined markers showed improved accuracy in a recent study on LOS [41]. More high-precision studies are needed to examine these markers with an emphasis on sensitivity and negative predictive value (NPV).

## Conclusion

The results of our study support an association between NLR values and the development of sepsis among neonates. NLR represents a unique inflammatory marker whose elevation in neonatal sepsis implicates immune system imbalance in the pathogenesis of the disease. Further, our findings support NLR as a promising biomarker that can be readily integrated into clinical settings to aid in diagnosing neonatal sepsis. With the development of new biomarkers and therapeutic modalities, we can better prevent and treat neonatal sepsis to decrease long-term morbidity and mortality. The NLR, as an inflammatory biomarker, measures innate-adaptive immune system balance. As evidenced by our results, restoring balance to the innate and adaptive immune system may serve as attractive therapeutic target. Theoretically, a reduction in NLR values could be used to measure therapeutic efficacy, reflecting the restoration of balance within these systems.

## Data Availability

The dataset supporting the conclusions of this article is included within the article.

## Abbreviations

NLR: Neutrophil to lymphocyte ratio
ICU: Intensive care unit
SMD: Standardized mean difference
95% CI: 95% Confidence interval
CRP: C-reactive protein
N: Number
LOS: Late-onset sepsis
EOS: Early-onset sepsis
NOS: The Newcastle-Ottawa Quality Assessment Scale
R: Retrospective
P: Prospective
GBS: Group B Streptoccoci
PRISMA: Preferred Reporting Items for Systematic Reviews and Meta-Analyses
WMD: Weighted mean difference
SROC: Summary receiver operating characteristic
DOR: Diagnostic odds ratio
I/T: Immature to total neutrophil
